# Serum TRPA1 mediates the association between olfactory function and cognitive function

**DOI:** 10.3389/fnagi.2024.1411031

**Published:** 2024-06-10

**Authors:** Xiaoniu Liang, Zhenxu Xiao, Jie Wu, Xiaoxi Ma, Qianhua Zhao, Ding Ding

**Affiliations:** ^1^Institute of Neurology, Huashan Hospital, Fudan University, Shanghai, China; ^2^National Clinical Research Center for Aging and Medicine, Huashan Hospital, Fudan University, Shanghai, China; ^3^National Center for Neurological Disorders, Huashan Hospital, Fudan University, Shanghai, China

**Keywords:** cognitive function, olfactory identification, TRPA1, mediation analysis, elderly

## Abstract

**Background:**

Olfactory dysfunction was associated with poorer cognition. However, the association between transient receptor potential cation channel subfamily A member 1 (TRPA1) and cognitive function have not been studied. This study aimed to evaluate the mediation effect of TRPA1 on the association between olfactory and cognitive function among Chinese older adults.

**Methods:**

We recruited 121 participants with cognitive impairment (CI) and 135 participants with normal cognition (NC) from a memory clinic and the “Shanghai Aging Study.” Olfactory identification of each participant was measured by the Sniffin’ Sticks Screening Test 12 (SSST-12). Serum TRPA1 were quantified using the Enzyme-Linked Immunosorbent Assay. The mediation effects of TRPA1 on the association between olfactory function and cognitive function were explored using mediation analysis.

**Results:**

The CI group had a significantly higher proportion of the high level of serum TRPA1 (58.7%) than the NC group (42.2%) (*p* = 0.0086). After adjusted for gender, age, and years of education, mediation analysis verified that TRPA1 partially mediated the association between SSST-12 and Mini Mental State Examination (MMSE). It also verified that TRPA1 partially mediated the association between the identification of peppermint and MMSE.

**Conclusion:**

Our study emphasizes the mediation role of TRPA1 in the relationship between olfactory and cognitive function among older adults. Further research is necessary to explore the mechanism of TRPA1 on the relationship between olfactory and cognitive decline.

## Introduction

Olfactory dysfunction affects 24–75% of older adults, with its prevalence substantially increasing by age (Choi et al., 2018). Olfactory dysfunction is an important clinical symptom indicating an early stage of neurodegenerative diseases, including Alzheimer’s disease (AD), frontotemporal lobar degeneration, dementia with Lewy bodies, and Parkinson’s disease (Attems et al., 2014). Olfactory identification is likely to involve the most elaborate network of brain regions associated with language processing, semantic and episodic memory, as well as olfactory perception (Wilson et al., 2006). Several epidemiologic studies demonstrated that poorer olfactory function was significantly associated with poorer cognition in population-based cohorts (Stanciu et al., 2014; Devanand et al., 2015; Liang et al., 2016; Roberts et al., 2016; Yaffe et al., 2017).

Our previous cross-sectional study showed that participants with mild cognitive impairment (MCI) performed worse olfactory function, especially the ability to identify peppermint among 12 odors in the Sniffin’ Sticks Screening Test 12 (SSST-12), than those with normal cognition (NC) (Liang et al., 2016). Our cohort study showed that an inability to smell peppermint was related to a higher risk for incident dementia, and was inversely associated with the annual rate of change in the Mini Mental State Examination (MMSE) score (Liang et al., 2020).

Peppermint was found to enhance memory (Moss et al., 2009) and the sustained visual attention task (Warm et al., 1991). Peppermint was mainly composed of L-menthol, carboxyl esters, menthone, menthyl acetate (Thomson Healthcare, 2007) and other components (Leung, 1980). The main cellular receptor for L-menthol is transient receptor potential cation channel subfamily A member 1 (TRPA1), which is associated with AD (Lee et al., 2016; Bosson et al., 2017). Studies have shown that the protein expression of TRPA1 channel significantly increased in AD transgenic mice, mainly in astrocytes of the hippocampus, and TRPA1 could be involved in the pathogenesis of AD through multiple pathways (Lee et al., 2016; Bosson et al., 2017; Paumier et al., 2022).

In this pilot study, we aimed to evaluate the potential mediation effect of TRPA1 on the association between olfactory and cognitive function in Chinese older adults.

## Materials and methods

### Study participants

Participants with cognitive impairment (CI) were from the memory clinic of the department of neurology, Huashan Hospital, Shanghai, China from December 2020 to May 2021. Participants met the inclusion criteria if they (1) visited the memory clinic due to memory complaints from themselves or the proxy; (2) were diagnosed with AD clinical syndrome or MCI; (3) had the ability to cooperate with neuropsychological tests and physical examinations; (4) completed the olfactory assessment; (5) 60 years or older; and (6) agreed to the blood draw.

The Shanghai Aging Study (SAS) is a community-based cohort in downtown Shanghai, China. SAS aimed to explore the prevalence, incidence, and risk factors for MCI and dementia among older residents. The detailed recruitment procedure and study design have been published elsewhere (Ding et al., 2014). In the SAS, the participants were diagnosed with NC from the third wave of follow-up between June 2020 and November 2021 if they (1) completed the olfactory assessment; (2) had the ability to cooperate with neuropsychological tests and physical examinations; (3) 60 years or older; and (4) agreed to the blood draw.

We excluded participants who (1) had histories of rhinal or paranasal sinuses diseases; (2) underwent maxillofacial surgery; (3) had chronic sinusitis, asthma, chronic obstructive pulmonary disease, or acute upper respiratory tract infection within 7 days before the olfactory assessment; and (4) alcohol or drug abuse.

### Demographics, lifestyles, and medical history

The lifestyle and demographic characteristics were collected from the participants and/or proxy through questionnaires, including gender, age, and years of education. Hypertension and diabetes mellitus were confirmed by the medical records (Ding et al., 2014).

### Olfactory identification test

Olfactory identification was a procedure in which a participant needed to accurately identify olfactory stimuli using alternative choices. Olfactory identification test was assessed by the SSST-12, which includes 12 common odors (orange, cinnamon, leather, banana, peppermint, liquorice, lemon, cloves, coffee, rose, pineapple, and fish) presented on felt-tip sticks (Hummel et al., 2001). The SSST-12 was devised by G. Kobal in Erlangen, Germany, and it is a portable, rapid (approximately 6 min), suited for inexpensive and repetitive screening of olfactory identification. The administrator of SSST-12 was blind for the cognitive diagnosis of each participant. Before the test, participants were reminded to stay away from chewing sweets, cigarettes or gum. Testing was performed in an air-conditioned and quiet room. A brief history was recorded, including questions related to the participant’s previous diseases, olfactory experience, occupation, drug intake and smoking habits. When presenting the odors, the administrator was wearing cotton gloves. The opened odor sticks were positioned about 2 cm in front of both nostrils of each participant. Participants were then asked to sniff for no longer than 3–4 s and to choose one of four answers from a list that described the best odor. An interval of 30s was set between the different sticks. Detailed instructions of SSST-12 were reported elsewhere (Liang et al., 2016).

### Neurological, neuropsychological assessments, and consensus diagnosis

Comprehensive neuropsychological tests were administered by the certified psychometrists in accordance with the education level of each participant. All tests conducted had been translated, adapted, and validated within the local Chinese population.

Each participant from the memory clinic received a battery of neuropsychological tests including (Xiao et al., 2021): (1) Montreal Cognitive Assessment-Basic (MoCA-B); (2) MMSE; (3) Auditory Verbal Learning Test; (4) Boston Naming Test; (5) Rey-Osterrieth Complex Figure test; (6) Symbol Digit Modalities Test; and (7) Trail-making test A&B. For those who were unable or refused to complete the whole battery of tests, only MoCA-B and MMSE were administered. Because the MMSE is less sensitive for MCI detection (Nasreddine et al., 2005), MMSE was used together with MoCA-B to discriminate MCI and dementia.

As for the participants from SAS, a battery of similar neuropsychological tests was administered due to the study design of SAS (Zhang et al., 1990; Ding et al., 2015): (1) MMSE; (2) Auditory Verbal Learning Test; (3) Conflicting Instructions Task (Go/No Go Task); (4) Modified Fuld Object Memory Evaluation; (5) Modified Common Objects Sorting Test; (6) RMB (Chinese currency) test; (7) Stick Test; and (8) Trail-making test A&B.

Two study neurologists, one neuroepidemiologist and one neuropsychologist reviewed the medical, functional, neuropsychological, neurological, and psychiatric data and reached a consensus regarding the absence or presence of dementia using DSM-IV criteria (American Psychiatric Association, 1994). Probable AD was diagnosed using the NINCDS-ADRDA criteria (McKhann et al., 1984). Participants who met the criterion of probable AD were regarded as having AD clinical syndrome. The diagnosis of MCI was based on Petersen’s criteria (Petersen, 2004).

### Serum TRPA1 measurement

Blood was collected from the study participants. Serum and plasma samples were centrifuged, aliquoted, and stored at −80°C. Serum TRPA1 were quantified using the Enzyme-Linked Immunosorbent Assay test on the Enzyme labeling apparatus (352 Labsystems Multiskan MS), and serum samples were diluted at a 1:4 ratio following the manufacturer’s instructions. Duplicate measurements were taken for calibrators and quality controls. Sample measurements were conducted in a single run using kits with the identical lot numbers. Operators remained unaware of participants’ characteristics. The statistical median value of serum TRPA1 was used to categorize participants into low or high levels of serum TRPA1.

### Statistical analysis

Continuous variables were expressed as the mean (standard deviation) or median (lower quartile[25%], upper quartile[75%]), and categorical variables were expressed as frequencies (%). The Wilcoxon rank-sum test and Pearson chi-square test were used to compare continuous and categorical variables.

Scatter plots were performed to analyze the correlations between SSST-12 and serum TRPA1, between SSST-12 and MMSE, and between serum TRPA1 and MMSE. Their correlations were assessed using the generalized linear model with the adjustment for gender, age, and years of education.

The logistic regression model was used to detect the association between serum TRPA1, the level of serum TRPA1, SSST-12, olfactory dysfunction, or the identification of peppermint and CI after adjusted for gender, age, and years of education. Risk was presented as odds ratio (OR) and 95% confidence interval (95%CI).

Mediation analysis was conducted to decompose the total effect of olfactory function on cognitive function into a natural direct effect and a natural indirect effect through TRPA1 after adjusted for gender, age, and years of education.

Two-tailed tests were used to estimate all *p* values and 95% confidence intervals. Statistically significant differences were identified at *p* < 0.05. Mediation analysis was conducted using R packages “lavaan” and “mediation.” Data analysis was conducted in SAS 9.4 (SAS Institute Inc., Cary, NC, United States) and R Software (version 4.1.2).

## Results

### Characteristics of the participants

We recruited 256 participants (164 females and 92 males), including 135 participants with NC and 121 participants with CI. Table 1 displayed the characteristics of the study participants. Significant differences were observed in the level of serum TRPA1 (*p* = 0.0086), education year (*p* = 0.0002), MMSE (*p* < 0.0001), and SSST-12 (*p* < 0.0001) between the two groups. The CI groups had higher proportion of the high level of serum TRPA1 (58.7%), the lower median education year (median = 11), the lower median MMSE score (median = 27), and the lower median SSST-12 score (median = 7).

**Table 1:** Characteristics of study participants.

Clinical features	Total (*N* = 256)	Normal cognition (*N* = 135)	Cognitive impairment (*N* = 121)	*p*-value
Level of TRPA1, *n* (%)				
Low	128 (50.0)	78 (57.8)	50 (41.3)	**0.0086**
High	128 (50.0)	57 (42.2)	71 (58.7)	
TRPA1, pg./mL, mean ± SD	482.8 ± 88.0	472.6 ± 82.7	494.1 ± 92.5	0.0626
Gender, female, *n* (%)	164 (64.1)	89 (65.9)	75 (62.0)	0.5116
Age, year, median (Q1, Q3)	74 (71, 80)	73 (71, 78)	76 (71, 82)	0.0553
Education, year, median (Q1, Q3)	12 (9, 13)	12 (10, 15)	11 (9, 12)	**0.0002**
MMSE, score, median (Q1, Q3)	28 (26, 29)	29 (28, 30)	27 (25, 28)	**<0.0001**
SSST-12, score, median (Q1, Q3)	8 (6, 9)	8 (7, 9)	7 (5, 9)	**<0.0001**
Correct identification of peppermint, *n* (%)	210 (82.0)	115 (85.2)	95 (78.5)	0.2083

MMSE, mini-mental state examination; Q1, lower quartile; Q3, upper quartile; SSST-12, Sniffin’ Sticks Screening Test 12; SD, standard deviation; TRPA1, transient receptor potential cation channel subfamily A member 1.

### Association analysis

**Figure 1:**
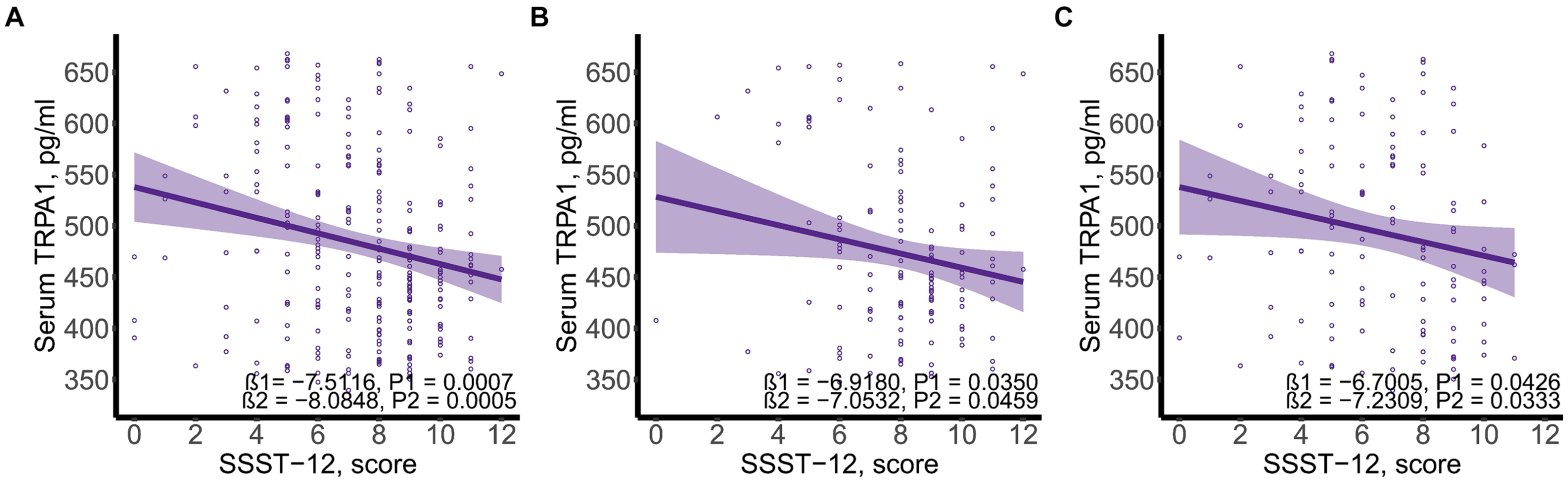
Scatter plots of serum SSST-12 and TRPA1. **(A)** total participants; **(B)** participants with normal cognition; **(C)** participants with cognitive impairment. β1 was the beta correlation coefficient. β2 was the beta correlation coefficient after adjusted for gender, age, and years of education. The purple area represented the 95% confidence interval. SSST-12, Sniffin’ Sticks Screening Test 12; TRPA1, transient receptor potential cation channel subfamily A member 1.

**Figure 2:**
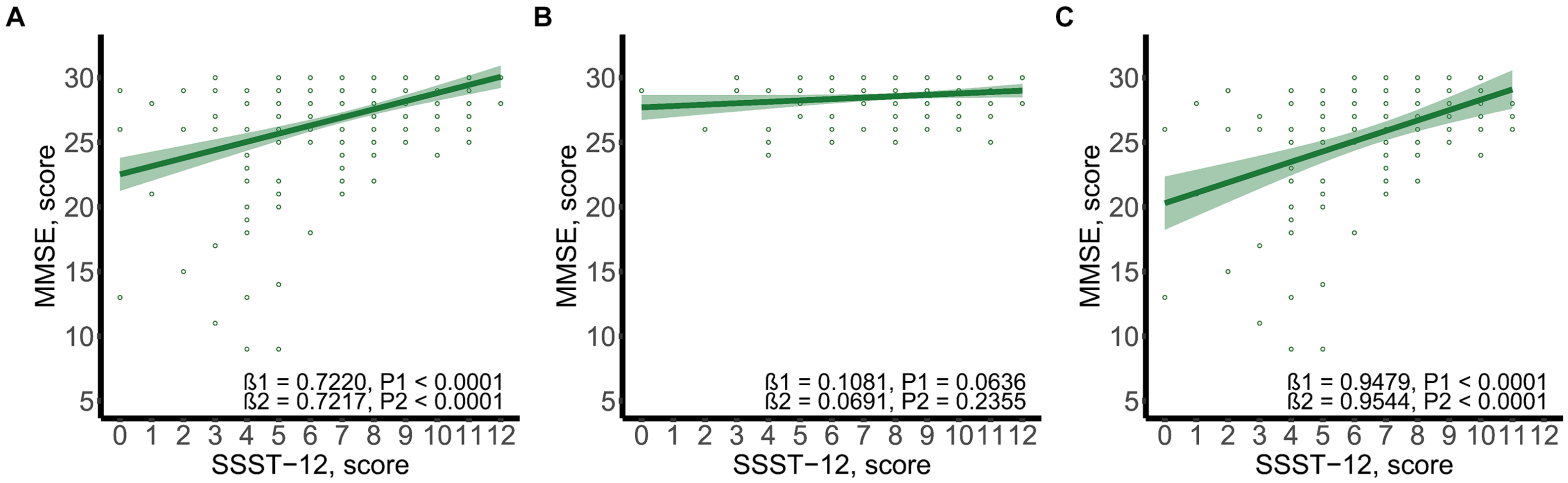
Scatter plots of SSST-12 and MMSE. **(A)** total participants; **(B)** participants with normal cognition; **(C)** participants with cognitive impairment. β1 was the beta correlation coefficient. β2 was the beta correlation coefficient after adjusted for gender, age, and years of education. The green area represented the 95% confidence interval. MMSE, mini-mental state examination; SSST-12, Sniffin’ Sticks Screening Test 12.

**Figure 3:**
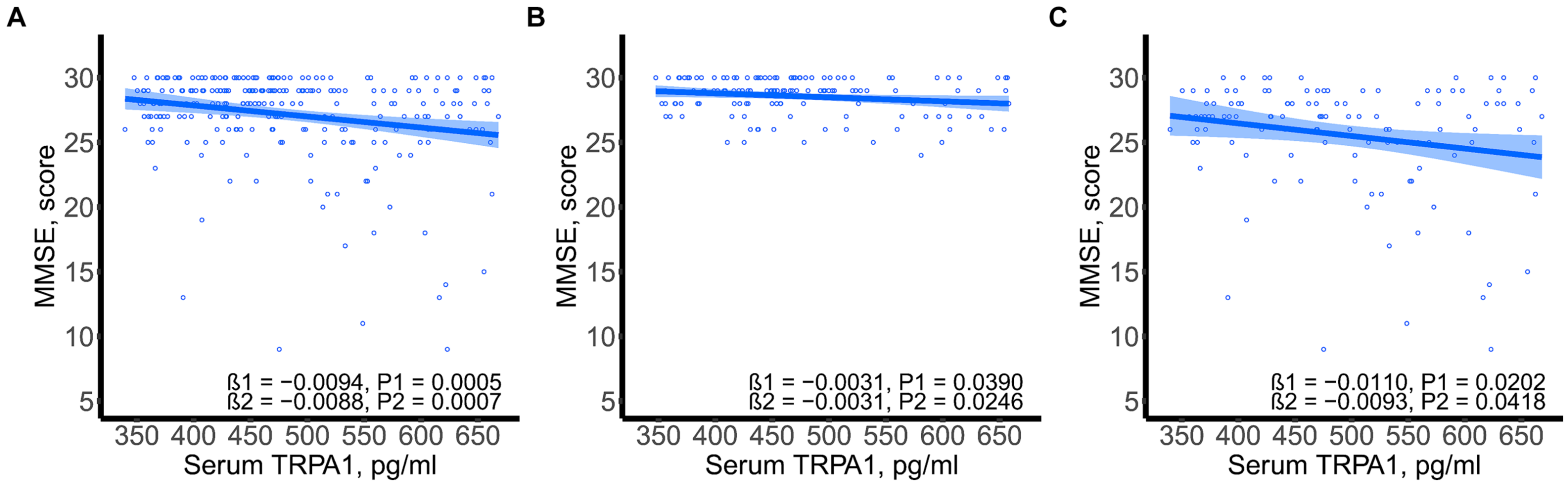
Scatter plots of serum TRPA1 and MMSE. **(A)** total participants; **(B)** participants with normal cognition; **(C)** participants with cognitive impairment. β1 was the beta correlation coefficient. β2 was the beta correlation coefficient after adjusted for gender, age, and years of education. The blue area represented the 95% confidence interval. MMSE, mini-mental state examination; TRPA1, transient receptor potential cation channel subfamily A member 1.

SSST-12 were inversely correlated with serum TRPA1 in total participants (β = −8.0848, 95%CI: −12.6423, −3.5272), participants with NC (β = −7.0532, 95%CI: −13.9778, −0.1286) and participants with CI (β = −7.2309, 95%CI: −13.8897, −0.5721) after adjusted for gender, age, and years of education (Figure 1). SSST-12 were positively correlated with MMSE in total participants (β = 0.7217, 95%CI: 0.5454, 0.8980) and participants with CI (β = 0.9544, 95%CI: 0.6528, 1.2559) after adjusted for gender, age, and years of education (Figure 2). The serum TRPA1 level was inversely correlated with MMSE in total participants (β = −0.0088, 95%CI: −0.0138, −0.0037) and participants with CI (β = −0.0093, 95%CI: −0.0182, −0.0003) after adjusted for gender, age, and years of education (Figure 3).

As shown in Table 2, the higher serum TRPA1 level was associated with an increased risk for participants with CI (OR = 1.889, 95%CI: 1.125, 3.172) after adjusted for gender, age, and years of education. Lower SSST-12 score (OR = 0.776, 95%CI: 0.685, 0.878) and olfactory dysfunction (OR = 3.116, 95%CI: 1.592, 6.101) were associated with an increased risk for participants with CI after adjusted for gender, age, and years of education. The correct identification of peppermint may be a protector for participants with CI (OR = 0.858, 95%CI: 0.428, 1.719), although the finding did not reach statistical significance.

**Table 2:** Odds ratios for serum TRPA1 between participants with normal cognition and cognitive impairment.

	OR (95%CI)	OR (95%CI)*
Serum TRPA1	1.003(1.000, 1.006)	1.003(1.000, 1.006)
Level of TRPA1		
Low	1	1
High	1.943(1.181, 3.196)	1.889(1.125, 3.172)
SSST-12	0.754(0.671, 0.847)	0.776(0.685, 0.878)
*Olfactory dysfunction*		
No	1	1
Yes	3.537(1.853, 6.751)	3.116(1.592, 6.101)
Peppermint		
Wrong	1	1
Right	0.661(0.346, 1.263)	0.858(0.428, 1.719)

* The OR (95%CI) was calculated by logistic regression after adjusted for gender, age, and years of education.

95%CI, 95% confidence interval; OR, odds ratio; SSST-12, Sniffin’ Sticks Screening Test 12; TRPA1, transient receptor potential cation channel subfamily A member 1.

### Mediation analysis

**Figure 4:**
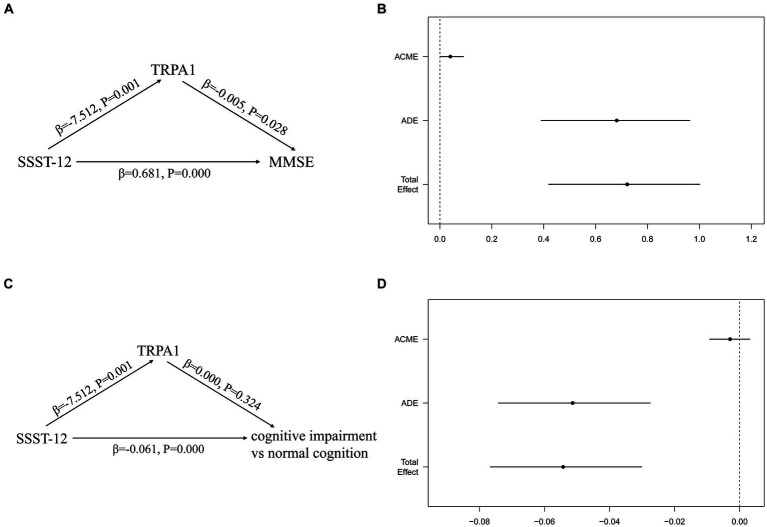
Mediation effects of the serum TRPA1 in the relationship between olfactory function and cognitive function. **(A)** the mediation effect of SSST-12 on MMSE via the serum TRPA1; **(B)** the mediation effect of SSST-12 on MMSE via the serum TRPA1 after adjusted for gender, age, and years of education; **(C)** the mediation effect of SSST-12 on cognitive impairment via the serum TRPA1; **(D)** the mediation effect of SSST-12 on cognitive impairment via the serum TRPA1 after adjusted for gender, age, and years of education. Results for figure B and D are presented as effect sizes (95% confidence interval) for the association of SSST-12 with MMSE and cognitive impairment. ADE: the effect of the SSST-12 on MMSE or cognitive impairment, not explained by the serum TRPA1. ACME: the effect of the SSST-12 on MMSE or cognitive impairment acting through the serum TRPA1. Total effect: the effect of the SSST-12 on MMSE or cognitive impairment. ACME, Average Causal Mediated Effect; ADE, Average Direct Effect; MMSE, mini-mental state examination; SSST-12, Sniffin’ Sticks Screening Test 12; TRPA1, transient receptor potential cation channel subfamily A member 1.

Figure 4 showed the total, direct and indirect effects for the mediating role of TRPA1 on the relationship between olfactory function and cognitive function in mediation models. As shown in Figure 4B, after adjusted for gender, age, and years of education, the estimated average causal mediated effect (ACME) (indirect effect estimate β = 0.0402, 95%CI: 0.0023, 0.0906), average direct effect (ADE) (direct effect estimate β = 0.6816, 95%CI: 0.3906, 0.9630), and total effects (total effect estimate β = 0.7217, 95%CI: 0.4193, 1.0015) were all statistically significant, suggesting a partial mediation effect of TRPA1 in the association between olfactory function and MMSE, and TRPA1 had the mediation effect with a proportion of mediation up to 5.57%.After adjusted for gender, age, and years of education, the estimated ADE (direct effect estimate β = −0.0513, 95%CI: −0.0741, −0.0275) and total effects (total effect estimate β = −0.0543, 95%CI: −0.0766, −0.0301) were statistically significantly different from zero, but the estimated ACME were not (Figure 4D). The results suggested that lower SSST-12 may be associated with higher serum TRPA1, which in turn made participants more likely to have worse cognitive function.

**Figure 5:**
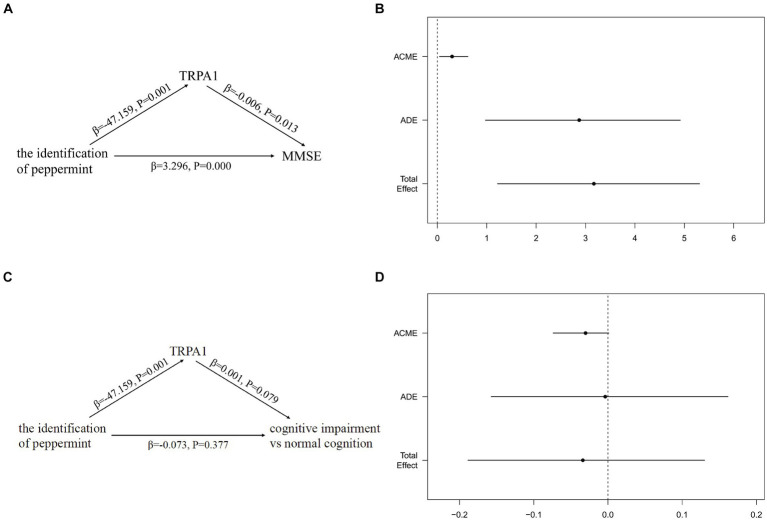
Mediation effects of the serum TRPA1 in the relationship between the identification of peppermint and cognitive function. **(A)** The mediation effect of the identification of peppermint on MMSE via the serum TRPA1; **(B)** the mediation effect of the identification of peppermint on MMSE via the serum TRPA1 after adjusted for gender, age, and years of education; **(C)** the mediation effect of the identification of peppermint on cognitive impairment via the serum TRPA1; **(D)** the mediation effect of the identification of peppermint on cognitive impairment via the serum TRPA1 after adjusted for gender, age, and years of education. Results for figure **(B,D)** are presented as effect sizes (95% confidence interval) for the association of the identification of peppermint with MMSE and cognitive impairment. ADE, the effect of the identification of peppermint on MMSE or cognitive impairment, not explained by the serum TRPA1. ACME, the effect of the identification of peppermint on MMSE or cognitive impairment acting through the serum TRPA1. Total effect, the effect of the identification of peppermint on MMSE or cognitive impairment. ACME, Average Causal Mediated Effect; ADE, Average Direct Effect; MMSE, mini-mental state examination; TRPA1, transient receptor potential cation channel subfamily A member 1.

Figure 5 showed the total, direct and indirect effects for the mediating role of TRPA1 on the relationship between the identification of peppermint and cognitive function in mediation models. As shown in Figure 5B, after adjusted for gender, age, and years of education, the estimated ACME (indirect effect estimate β = 0.2975, 95%CI: 0.0443, 0.6200), ADE (direct effect estimate β = 2.8716, 95%CI: 0.9727, 4.9200), and total effects (total effect estimate β = 3.1691, 95%CI: 1.2178, 5.3100) were all statistically significant, suggesting a partial mediation effect of TRPA1 in the association between the identification of peppermint and MMSE, and TRPA1 had the mediation effect with a proportion of mediation up to 9.39%. However, the estimated ACME, the estimated ADE and total effects were all not statistically significant (Figure 5D). The results suggested that the incorrect identification of peppermint may be associated with higher serum TRPA1, which in turn made participants more likely to have worse cognitive function.

## Discussion

This study marks the initial attempt to partition the effects of olfactory function on cognitive function into direct effects and indirect effects (mediated by serum TRPA1) among Chinese older adults. Mediation analysis of olfactory function (both SSST-12 and peppermint) with MMSE showed a partial mediation effect acting through serum TRPA1.

The association between SSST-12 and CI has been reported in a few epidemiologic studies. We previously demonstrated that lower SSST-12 score was related to MCI (OR = 1.19, 95%CI:1.11, 1.27) in the older adults by using the baseline data of SAS (Liang et al., 2016). Poor olfactory dysfunction (assessed by 16-item Sniffin’ Sticks identification test [SSST-16]) was significantly associated with an increased risk for non-amnestic MCI, amnestic MCI, and MCI (Dong et al., 2023). However, to our knowledge, the effect of TRPA1 on olfactory dysfunction or CI has not been reported in any population-based studies. TRPA1 is closely associated with inflammation, pruritus, and chronic pain, and TRPA1 is considered to be a promising treatment for them (Hu et al., 2023). Increased TRPA1 mRNA expression in whole blood cells was significantly related to decreased pain symptoms in chronic pain patients (Bell et al., 2014; Sukenaga et al., 2016). Epidemiologic studies have shown that patients with AD reported less pain (Scherder et al., 1999; Mantyselka et al., 2004; Achterberg et al., 2010; Jensen-Dahm et al., 2012), and patients with dementia were less likely to use analgesics (Horgas and Tsai, 1998; Morrison and Siu, 2000; Mantyselka et al., 2004).

Multiple pathways may implicate TRPA1 in the pathogenesis of AD mice. TRPA1 receptors mediate deteriorating effects in the decline of memory (Borbely et al., 2019). Functional ablation of the TRPA1 channel in mice improved hippocampal functions, demonstrating by reduced anxiety-like behavior, improved fear-related or spatial learning and memory, novel location recognition and social interactions (Lee et al., 2017). Astrocytic TRPA1 and GABA coordinately suppress hippocampal circuit function (Cheng et al., 2023). TRPA1 is expressed on astrocytes in the hippocampus, and the production of amyloid-β activates this channel, thereby initiated this hyperactivity and subsequently induced the hyperactivity of nearby neurons, which is a pivotal factor in the progression of AD (Bosson et al., 2017). The toxic effect of amyloid-β on astrocytes, triggered by TRPA1 channel activation, is crucial to the progression of AD, and TRPA1 blockade prevents irreversible neuronal dysfunction (Paumier et al., 2022). TRPA1-Ca^2+^-PP2B signaling may be crucial to regulate pathogenesis of AD and astrocyte-derived inflammation (Lee et al., 2016). Melatonin may be an effective option in the treatment and prophylaxis of AD by reducing cytosolic Ca^2+^ concentration, apoptosis and intracellular ROS through TRPA1 channels (Ozsimsek and Ovey, 2022). These studies showed that blocking TRPA1 could prevent irreversible neuronal dysfunction, and TRPA1 might be a potential therapeutic target for neuroprotection (Hu et al., 2023).

Several advantages existed in our study. Firstly, neuropsychological assessments and the diagnosis of CI were administered by the certified neurologists and neuropsychologists. Secondly, TRPA1 was tested among the patients with CI for the first time. Thirdly, we studied the mediating effect of serum TRPA1 on the causal pathway from olfactory function to cognitive function. Our findings warrant cautious interpretation in light of several limitations. The inherent cross-sectional design precludes establishing causal relationships, while the small sample size may have affected the detection of the mediation role of TRPA1 in the relationship between olfactory function and cognitive impairment. Future studies with larger samples are necessary to validate our findings. Moreover, as our study focused on older adults, further basic research is needed to confirm our results. Additionally, due to the absence of an established cutoff value for serum TRPA1, we utilized the statistical median to categorize participants into low or high levels of serum TRPA1, tailored specifically for this study. Despite the relative arbitrariness of this cutoff, our study still revealed a significant association.

In conclusion, our study explored the mediation role of TRPA1 in the relationship between olfactory and cognitive function among the Chinese older adults. Our findings provide preliminary evidence suggesting a mechanism linking olfactory and cognitive function, highlighting the potential significance of TRPA1 as a biomarker for cognitive impairment.

## Data availability statement

The datasets presented in this article are available from the corresponding author upon reasonable request and with permission of Huashan Hospital. Requests to access the datasets should be directed to DD, dingding@huashan.org.cn.

## Ethics statement

The studies involving humans were approved by Medical Ethics Committee of Huashan Hospital, Fudan University, Shanghai, China. The studies were conducted in accordance with the local legislation and institutional requirements. The participants provided their written informed consent to participate in this study.

## Author contributions

XL: Conceptualization, Data curation, Formal analysis, Funding acquisition, Investigation, Methodology, Project administration, Supervision, Validation, Visualization, Writing – original draft, Writing – review & editing. ZX: Conceptualization, Data curation, Investigation, Methodology, Writing – review & editing. JW: Investigation, Methodology, Visualization, Writing – review & editing. XM: Data curation, Methodology, Supervision, Writing – review & editing. QZ: Conceptualization, Funding acquisition, Methodology, Project administration, Resources, Visualization, Writing – review & editing. DD: Conceptualization, Funding acquisition, Investigation, Methodology, Project administration, Resources, Supervision, Validation, Visualization, Writing – review & editing.
