# Corrigendum: Serum TRPA1 mediates the association between olfactory function and cognitive function

**DOI:** 10.3389/fnagi.2024.1485432

**Published:** 2024-09-19

**Authors:** Xiaoniu Liang, Zhenxu Xiao, Jie Wu, Xiaoxi Ma, Qianhua Zhao, Ding Ding

**Affiliations:** ^1^Institute of Neurology, Huashan Hospital, Fudan University, Shanghai, China; ^2^National Clinical Research Center for Aging and Medicine, Huashan Hospital, Fudan University, Shanghai, China; ^3^National Center for Neurological Disorders, Huashan Hospital, Fudan University, Shanghai, China

**Keywords:** cognitive function, olfactory identification, TRPA1, mediation analysis, elderly

In the published article, there was an error in the **Funding** statement, where the funding source “Shanghai Municipal Health Commission Project (2020YJZX0101)” was erroneously omitted.

The correct **Funding** statement appears below.

